# Concise Review: Dissecting a Discrepancy in the Literature: Do Mesenchymal Stem Cells Support or Suppress Tumor Growth?

**DOI:** 10.1002/stem.559

**Published:** 2010-11-18

**Authors:** Ann H Klopp, Anshul Gupta, Erika Spaeth, Michael Andreeff, Frank Marini

**Affiliations:** a Department of Radiation Oncology, The University of Texas MD Anderson Cancer CenterHouston, Texas, USA; b Department of Leukemia, The University of Texas MD Anderson Cancer CenterHouston, Texas, USA

**Keywords:** Mesenchymal stem cells, Adipose stem cells, Cancer, Stroma, Microenviroment

## Abstract

The discovery that mesenchymal stem cells (MSCs) are recruited into tumors has led to a great deal of interest over the past decade in the function of MSCs in tumors. To address this, investigators have used a variety of tumor models in which MSCs are added exogenously to determine their impact on tumor development. Interestingly, many studies have reported contradicting results, with some investigators finding that MSCs promote tumor growth and others reporting that MSCs inhibit tumor growth. Many mechanisms have been reported to account for these observations, such as chemokine signaling, modulation of apoptosis, vascular support, and immune modulation. In this review, we analyzed the differences in the methodology of the studies reported and found that the timing of MSC introduction into tumors may be a critical element. Understanding the conditions in which MSCs enhance tumor growth and metastasis is crucial, both to safely develop MSCs as a therapeutic tool and to advance our understanding of the role of tumor stroma in carcinogenesis. Stem Cells 2011;29:11–19

## INTRODUCTION

Mesenchymal stem cells or multipotent mesenchymal stromal cells (MSCs) are multipotent progenitor cells that exhibit a marked tropism for tumors. MSCs form a tumor's fibrovascular network, differentiating into tumor-associated fibroblasts (TAFs) and vascular pericytes [1–3]. Despite extensive investigations over the past 5 years, the impact of unmodified MSCs on tumor progression remains unclear. Many studies have shown that MSCs promote tumor progression and metastasis while other studies report that MSCs suppress tumor growth (referenced in Tables 1 and 2). The reason for this discrepancy is unknown, but it may be attributable to differences in tumor models, the heterogeneity of MSCs, the dose or timing of the MSCs injected, the animal host, or another factor that is not yet appreciated.

**Table 1:** Studies reporting that MSC promote tumor growth

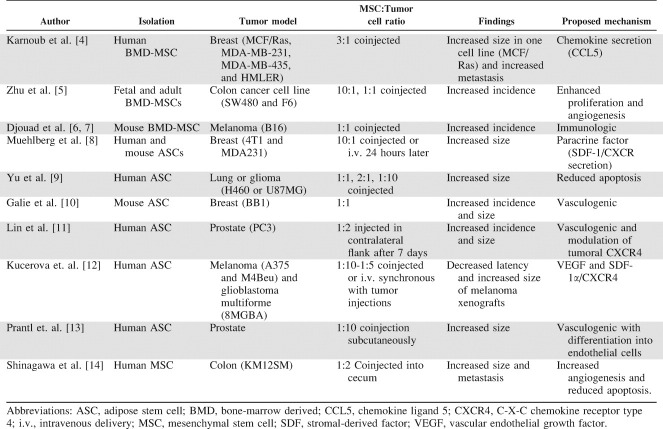

**Table 2:** Studies reporting that mesenchymal stem cells inhibit tumor growth

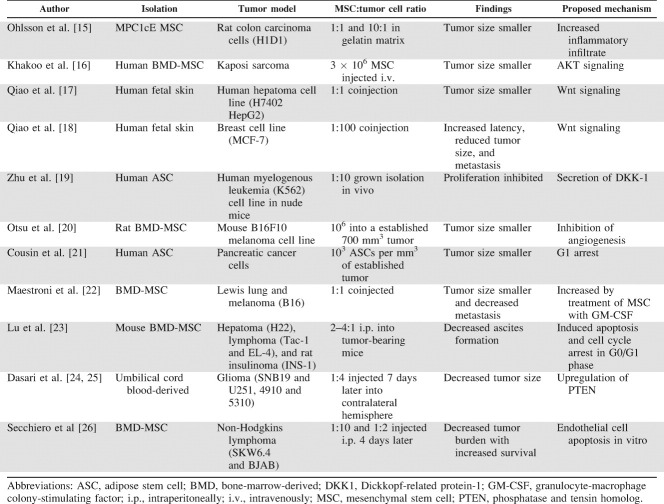

The tropism of MSC for tumors makes MSCs uniquely destined to function as cellular delivery vehicles for antitumor agents [27–32]. MSCs can deliver a diverse array of agents, including interferon (IFN)-β, cytosine deaminase, tumor necrosis factor-related apoptosis-inducing ligand, and oncolytic viruses. These approaches have been explored in preclinical cancer models and yielded potent antitumor effects [29–35]. Tumor-targeted production of antitumor agents is likely to overcome any endogenous tumor-promoting effects of MSCs. However, it is important to determine under what conditions MSCs enhance tumor growth and metastasis to develop therapeutic applications and to understand the role of stroma in tumorigenesis. This review article focuses on studies that directly investigated the effects of MSCs on tumors in vivo and attempts to shed light on the contradictions reported in the literature.

### Isolation of MSCs

MSCs have been isolated from many types of adult and fetal tissues using similar methodologies [36]. Bone marrow and adipose tissues are rich sources of MSCs [37, 38]. MSCs have also been isolated from many other adult tissues, including kidney, skin, and the parathyroid gland [38–40]. MSCs or MSC-like cells have also been isolated from fetal tissues, including the skin, umbilical cord, and placenta [33, 41–43].

These tissue-derived MSCs share a number of important characteristics with bone marrow-derived MSCs, including cell surface marker expression, plastic adherence, and the capacity to differentiate into cells of mesenchymal lineage (i.e., fat, bone, muscle, and cartilage) under appropriate conditions [37]. In addition, MSCs demonstrate a tumor tropism that distinguishes them from other mesenchymal cells, such as differentiated fibroblasts [31, 44]. However, tissue-derived MSCs are likely to differ in critical ways from the well-studied bone marrow-derived MSCs. It is possible that the contradictions in study findings are attributable to the variability in MSCs from different sources (i.e., bone marrow-derived MSCs vs. tissue-derived MSCs) and variability in MSCs from donor to donor even when obtained from the same source using the same technique.

### Endogenous Functions of MSCs

MSCs are thought to endogenously support wound healing and hematopoiesis, but many of the native functions of MSCs remain poorly understood. When engrafted at sites of tissue injury, MSCs differentiate into connective tissue elements, support vasculogenesis, and secrete cytokines and growth factors that facilitate healing. The potential of MSCs to promote tissue repair is being investigated in a diverse array of diseases, including ischemic heart disease, diabetes, and Parkinson's disease [45].

The function of MSCs in tumors is likely to parallel the role of MSCs in wound healing. As in wounds, MSCs differentiate in tumors into fibroblasts and pericytes and, perhaps, endothelial-like or vessel-attached cells [3, 46]. Additionally, MSCs secrete a number matrix proteins and cytokines that can increase proliferation and support vasculogenesis, such as vascular endothelial growth factor (VEGF) and platelet-derived growth factor (PDGF) [47].

MSCs also have complex immunomodulatory effects. MSCs can counteract inflammation, by suppressing host immune responses and preventing fibrosis. The immunosuppressive properties of MSCs are being exploited in an effort to reduce the toxicity of allogeneic bone marrow transplant due to graft versus host disease (GvHD) in which the transplanted cells formulate an immune response against the transplant recipient [48, 49].

## IMPACT OF MSC ON IN VIVO TUMORS

### MSC Promotion of Tumor Growth

Studies reporting that MSCs can promote tumor growth are listed in Table 1. Karnoub et al. [4] coinjected bone marrow-derived human MSCs with green fluorescent protein-labeled human breast cancer cells (MCF/Ras, MDA-MB-231, MDA-MB-435, and HMLER) in a ratio of 3:1 into immunocompromised mice. The MSCs accelerated tumor growth in one of the four cell lines (MCF/Ras) but did not affect local tumor growth in the other cell types. Coinjection with MSCs increased the number of breast cancer metastases that formed in all cell lines investigated.

Bone marrow-derived MSCs have been shown to increase the in vivo growth of colon cancer, lymphoma, and melanoma cells [5, 6, 50]. Adult- and fetal-derived MSCs were coinjected with colon cancer cells (SW480 and F6) in a murine xenograft model [5], resulting in an increased incidence of tumors with enhanced vascularity and necrosis. Both adult and fetal MSCs had similar growth-promoting effects, but adult MSCs appeared to favor tumor incidence more than fetal MSCs.

B16 melanoma cells transplanted into allogeneic mice did not form tumors unless MSCs were coinjected [6]. This finding suggests MSCs have immunosuppressive effects, which were required for tumor initiation in this model. MSCs also support the in vitro survival of follicular lymphoma B cells derived from human tumors. This protective effect of MSCs was augmented further by treating MSCs with tumor necrosis factor-α and lymphotoxin-α1β2 [50].

Adipose tissue is a rich source of multipotent MSCs (referred to as adipose stem cells [ASCs]), which exhibit tumor tropism and are thought to be functionally similar to bone marrow-derived MSCs [40]. Muehlberg et al. [8] demonstrated that ASCs can promote tumor growth in a syngeneic mouse model. Mammary breast cancer cells were cotransplanted with ASCs, resulting in the development of larger and more rapidly forming tumors [51]. Human ASCs have also been shown to favor tumor cell growth in nude mice. When human ASCs were coinjected subcutaneously with lung cancer or glioma cells (H460 or U87MG) into nude mice, there was a substantial increase in the tumor size and the number of viable tumor cells [9]. Lung cancer and Kaposi sarcoma xenografts were promoted by injection of white adipose tissue adjacent to tumor xenografts [52]. Adipose tissue-derived stromal cells were detected within these xenograft tumors, demonstrating that adipose stromal cells promote tumor growth when recruited from regional adipose tissue.

However, in most of these reports MSCs were added to fixed numbers of tumor cells, resulting in increased total numbers of cells injected including both tumor cells and MSCs. We have demonstrated earlier that MSCs proliferate in the presence of tumor cells in vivo, as evidenced by BrdUrd incorporation in vivo, whereas MSCs implanted without tumors did not proliferate [32]. Hence, the increased tumor mass observed could be related to: (a) increased number of tumor cells; (b) increased proliferation of MSCs in tumors; (c) combination of both. As the relative proportion of stroma cells in these tumors has never been determined, the observed “enhancement” of tumor growth has to be interpreted with caution.

Finally, MSCs have been reported to undergo transformation into malignant cells and form tumors in vivo. However, three groups of investigators have now found that the “malignant” MSCs were contaminated with tumor cells used for other projects in their laboratories. Garcia-Castro and coworkers reported initially that MSCs underwent spontaneous transformation but recently found contamination with HT1080 sarcoma cells [53, 54]. Rosland et al. reported a similar finding [55] but found recently that their MSCs were contaminated with HT1080 and U-2 OS cells [15]. These findings emphasize the importance of frequently subjecting cell lines to DNA fingerprinting, preferentially by short tandem repeat profiling and suggest caution when MSCs are reported as causing tumors or enhancing tumor growth. Importantly, after transplantation of MSCs into over 1,000 patients, not a single case of MSC-related tumors has been reported.

### MSC Inhibition of Tumor Growth

In direct contradiction to the studies reporting that MSCs support tumor growth, other studies have shown that MSCs suppress tumor growth (Table 2). MSCs inhibited the growth of rat colon carcinoma when coinjected with equal number of MSCs and tumor cells or with 10-fold more MSCs [56]. Macrophage and granulocyte infiltration was noted to be much higher in tumors injected with MSCs than in tumors not injected with MSCs, suggesting that MSCs had proinflammatory effects in this model. In fact, MSCs alone also generated increased engraftment of monocytes and granulocytes, which may have been a consequence of the immunogenicity of the MSCs transplanted into a nonidentical rat strain and may have contributed to the antitumor effect of MSCs in this model. In a highly inflammatory and angiogenic Kaposi sarcoma model, MSCs but not human umbilical vein endothelial cells, inhibited tumor growth in the xenografts [16]. The use of athymic nude mice in these experiments suggests that the inhibitory effects of MSCs were not due to immunomodulatory effects.

Human fetal skin-derived MSCs inhibited human liver cancer cell lines, with reduced proliferation, colony formation, and oncogene expression both in vitro and in vivo [17]. When these cell lines were coinjected with the same number of MSCs, tumor development was delayed and tumor size decreased.

The same fetal skin-derived MSCs inhibited growth of MCF-7 breast cancer cells in vitro [18]. The researchers found that treatment with conditioned media resulted in downregulation of survival factors, such as β-catenin, c-Myc, and survivin. This effect was mediated by an inhibitor of β-catenin signaling, Dickkopf-related protein-1 (DKK-1), which is secreted by MSCs [18]. The DKK-1 effects were suppressed in MSCs with the use of a neutralizing antibody and small interfering RNA, eliminating the growth inhibitory effects of MSCs.

Adipose-derived MSCs were also found to inhibit proliferation of primary leukemia cells [19]. This effect was mediated by secreted DKK-1, which was regulated by the stem cell transcription factor NANOG [19].

MSCs have been shown to suppress pancreatic tumors by altering cell cycle progression. In vitro coculture with MSCs increased rates of G1-phase arrest in pancreatic cancer cells [21]. In vivo injection of adipose-derived stem cells into established pancreatic cancer xenografts inhibited tumor growth [21]. In a similar approach, bone marrow-derived MSCs were injected into established subcutaneous melanomas, resulting in apoptosis and abrogation of tumor growth [20]. When MSCs were placed in a Matrigel insert, such that tumors were exposed to soluble factors but not in contact with MSC, MSCs had no cytotoxic effects.

The impact of hyperthermic treatment was tested in an in vitro study in which adipose-derived stem cells were heated [57]. Ovarian cancer SKOV-3 cells were cultured with supernatant from these heat-treated MSCs, resulting in decreased cell number and viability. Cho et al. [57] proposed that the decrease in cell number and viability was due to various secreted factors, including angiogenin, insulin-like growth factor (IGF) binding protein 4, neurotrophin 3, and chemokine (C–C motif) ligand (CCL) 18 [57].

## MECHANISMS OF MSC-MEDIATED EFFECTS OF TUMOR SUPPORT OR SUPPRESSION

### Vascular Support

MSCs have been reported to support the tumor vasculature directly, by differentiating into pericytes and perhaps endothelial cells [58] and through indirect mechanisms, by secreting vasculogenic growth factors [59]. Two lines of evidence suggest that MSCs differentiated into pericytes. Transplanted MSCs engraft in the perivascular niche in close contact with the underlying endothelial cells [60]. In addition, pericytes isolated from the stromal-vascular compartment have been shown to contain a MSC-like population with characteristic cell surface marker expression (CD10, CD13, and CD90) and the capacity to differentiate into tissues of mesenchymal lineage [61, 62]. Within mouse brain, a population of MSC-like cells has been identified in the perivasculature [63].

MSCs also secrete various proangiogenic factors, such as VEGF, fibroblast-derived growth factor, PDGF, and stromal-derived factor-1 (SDF-1). These cytokines promote endothelial and smooth muscle migration and proliferation at the tumor site, facilitating angiogenesis [64, 65].

MSC-secreted factors, such as VEGF, support blood vessel growth. MSCs expressing VEGF increased microvessel density in pancreatic xenografts [47]. However, recombinant VEGF at the same concentration found in the MSC-conditioned media did not have the same proliferative effect on vessel growth as did the MSC-conditioned media [65], suggesting that other proangiogenic cytokines are also involved. Other growth factors implicated in MSC effects on tumor vasculature include hepatocyte growth factor, cyclooxygenase, IGF-1, PDGF-α, and transforming growth factor-α1 [47]. Interestingly, expression of proangiogenic factors is increased by growth of MSCs as spheroids as compared with growth as monolayers [64].

In what appears to be a direct conflict with this data, MSCs appear to inhibit capillary growth under certain conditions. MSCs migrate in vitro toward endothelial cell-derived capillaries in Matrigel, incorporate into these vessels, and produce reactive oxygen species, resulting in endothelial cell apoptosis [20]. These researchers found that an endothelial cell to MSC ratio of 1:1 or 1:3 resulted in cytotoxicity but coinjection with 10% MSC resulted in no cytotoxicity. The growth-suppressing effect of MSCs was observed in vivo in which established melanomas injected with MSCs had reduced tumor growth and tumors exhibited lower vascular density [20].

### Fibrovascular Network

Fibroblasts, which are the primary cell type comprising tumor stroma, are a critical component of the tumor microenvironment. Tumor fibroblasts (referred to as TAFs) are derived in part from MSCs that may be recruited regionally or from circulating populations [44, 66]. After exposure to the tumor microenvironment, MSCs acquire expression of TAF antigens, such as α-smooth muscle actin, fibroblast-specific protein, vimentin, and SDF-1 in vivo and in vitro following coculture with tumor cells or using tumor-conditioned media [3, 66].

The importance of TAFs in promoting tumorigenesis has been well established in multiple tumor models [67]. TAFs extracted from human tumors facilitate the growth of human breast and ovarian cancers when coinjected into immunosuppressed mice [68]. This appears to involve multiple mechanisms, including the inhibition of cancer cell apoptosis, increased tumor cell proliferation, and promotion of angiogenesis. Thus, the acquisition of a TAF phenotype in MSC-derived tumor stroma provides indirect evidence for the protumor effect of MSCs.

### Immunomodulatory Effects of MSCs in Tumors

MSCs are generally thought to have immunosuppressive effects, which may be an important mechanism through which MSCs promote tumor growth or increase incidence of tumor formation in vivo. MSCs can directly impair the function of a variety of immune cells, including B and T lymphocytes, dendritic cells, and natural killer cells [69–75]. These immunosuppressive qualities of MSCs have been exploited to reduce GvHD following allogenic stem cell transplantation [76, 77].

MSCs suppress T-cell proliferation through multiple mechanisms. IFN-γ directly enhances the T-cell suppressive effects of MSCs through upregulation of an inhibitory cell surface marker, B7-H1 [78]. A subpopulation of MSC, identified by Stro-1+ expression, has the most potently inhibitory effects on T-cell proliferation [79].

Toll-like receptor (TLR) signaling has recently been shown to regulate the immunomodulatory properties of MSCs [80]. TLR respond to “danger” signals triggering the innate and adaptive immune responses. MSC express TLR and activation of certain receptors can polarize MSC to switch from a predominately immune suppressive to a proinflammatory phenotype [80]. It is possible that discrepant reports in the literature may be attributed to the activation of different TLR as a consequence of variability in TLR expression and environmental ligand expression.

The immunomodulatory effects of MSCs, if any, are not well understood within tumors. Djouad et al. [6] reported that the immunosuppressive action of MSCs led to a higher incidence of melanoma formation in a mouse model. Evidence for the effect of MSCs on tumor immunology also comes from a clinical study conducted to investigate whether the immunosuppressive qualities of MSCs could reduce the immunologic side effects of GvHD. Cotransplantation of MSCs has been reported to reduce the incidence of GvHD, but delivery of MSCs was also associated with higher rates of leukemia relapse in one study [77]. These results suggest that MSCs may have suppressed the graft versus leukemia effect and the graft versus host response. Alternatively, MSCs may have directly supported leukemia progression through other mechanisms, for example, by generating a specialized bone marrow niche supportive of residual leukemic cells [81, 82]. Clinical studies of the potential immunosuppressive effects of MSCs in Crohn's disease are in progress.

### Metastasis

Karnoub et al. reported that MSC-secreted CCL5 induced a transient prometastatic effect on breast cancer cells [4]. Tumors coinjected with MSCs exhibited a twofold to sevenfold increase in the number of breast cancer cells in the lungs [4]. Tumor cells isolated from the lung metastasis had equal rates of metastasis as compared with breast cancer cells taken from the primary tumors, suggesting that the effect of MSCs on metastasis is primarily not due to selection of a population of cells with an increased capacity to metastasize but rather to transient changes elicited by exposure to MSCs. Additionally, the prometastatic effect required the MSCs to be injected together with the tumor cells and was not seen when MSCs were injected distantly, demonstrating that the effect requires contact or exposure to paracrine signaling from the MSCs. Furthermore, MSCs themselves were not found in the pulmonary metastasis. These experiments support a model in which MSC-secreted factors reversibly modify tumor cells to increase their metastatic potential. The researchers additionally found that there was no increase in the number of metastatic tumor cells seen in the murine lungs when tumor cells were coinjected with other cells of mesenchymal lineage, suggesting that the prometastatic effect is a unique quality of MSCs. CCL5 secreted by MSCs and chemokine receptor 5 expression on breast cancer cells was found to be critical for the prometastatic effect of MSCs in two of the four breast cancer cell lines tested [4].

MSCs may modulate the epithelial-to-mesenchymal transition (EMT), a developmental process that is subverted by tumor cells resulting in a more invasive phenotype [83]. In breast cancer, coculture with MSCs resulted in upregulation of EMT-specific markers (N-cadherin, vimentin, Twist, and Snail) and a decrease in E-cadherin [84]. A similar effect was observed in prostate cancer cells that were cocultured with carcinoma-associated fibroblasts [85]. We observed that MSC-secreted factors increase mammosphere formation in normal and malignant breast cells and that mammospheres formed in the presence of MSC-conditioned media exhibited lower levels of E-cadherin expression and an increase in N-cadherin expression, characteristic of EMT [86]. This may be an additional mechanism by which MSCs influence tumor cell metastasis.

MSCs may also modify the metastatic niche, supporting the formation of early metastasis through vasculogenesis or growth factor secretion. For example, Corcoran et al. [87] reported that MSCs facilitated the entry of breast cancer cells into the bone marrow through Tac-1 regulation of SDF-α1 and C-X-C chemokine receptor type 4 (CXCR4), a G-protein couple receptor.

### Secretion of Paracrine Factors

MSCs secrete a variety of growth factors that are known to influence tumor proliferation, migration, and angiogenesis (Table 3). In addition, MSCs have also been recently reported to secrete exosomes or microparticles [94, 95]. Microparticles are lipid vesicles that are less than 1 mm in diameter and are secreted by cells. They may contain proteins or RNA that regulate intracellular signaling in adjacent cells [94, 95]. MSC-secreted microparticles contain microRNAs in the precursor form, which represent a subset of microRNAs found within MSCs. MSC-secreted microparticles may thus be one of the unappreciated mechanisms of MSC signaling within the tumor microenvironment.

**Table 3:** Proteins reported to be secreted by MSC

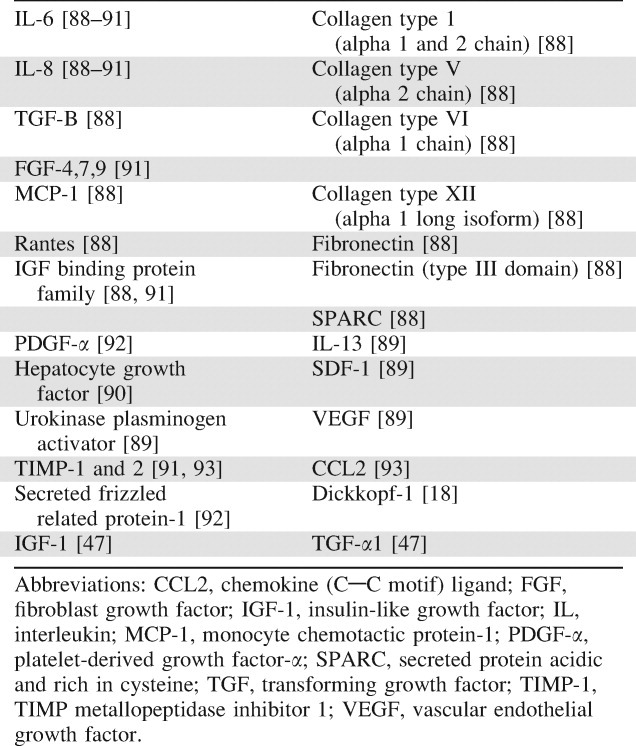

### Modulating Tumor Response to Cytotoxic Therapy

Although a great deal of research has focused on the role of MSCs as delivery vehicles for antitumor proteins or viruses, targeting endogenous MSCs in tumors also has the potential to be an effective therapy. Studies investigating the effects of MSCs on response to cytotoxic therapy have demonstrated that MSCs can protect tumor cells from chemotherapy. Interactions with MSCs in bone marrow has been shown to promote survival of acute and chronic myelogenous leukemia [82, 96–98]. Coculturing MSCs with acute myelogenous leukemia cells resulted in the upregulation of antiapoptotic bcl-2 with reduced rates of apoptosis in response to cytotoxic chemotherapy [82]. MSC-derived SDF-1 binds CXCR4, which is expressed on leukemic stem cells. Disrupting this interaction with CXCR4 inhibitors [98] overcomes, in part, the stroma-mediated chemotherapy resistance [82, 97].

MSCs secrete high levels of leptin, which has antiapoptotic properties, during adipocyte differentiation [99]. MSC-derived adipocytes decrease leukemic cell apoptosis in response to doxorubicin through a cell contact-dependent mechanism.

## CONCLUSION

MSCs interact with tumor cells in a myriad of ways, which have the potential to support or suppress tumor growth. Additionally, MSCs interact with their tumor-resident neighbors, such as immune cells (macrophages and T cells) and endothelial cells. Other cell types have also been shown to play roles in modulating tumor progression, thereby potentially exacerbating or inhibiting the outcomes of the MSC-tumor interactions.

The heterogeneity in MSC is likely a major factor contributing to the inconsistent reports about the effects of MSC in tumors. The MSC used in the studies reviewed here were isolated largely on the basis of plastic adherence. These MSCs exhibited appropriate cell surface marker expression as well as multipotency and yet these cell populations are fundamentally different in critical ways. To address this concern, cell surface markers that permit isolation of a more homogenous population are needed. Several markers, such as NG2 [100], have been proposed to be useful makers to identify a more homogenous population of MSC, which could make the results of studies such as these more readily comparable.

The studies reviewed here reported both growth promotion and suppression for the same cell types, including adult- and fetal-derived bone marrow, suggesting that the age of the donor does not determine the effect of MSC on tumor progression. Zhu et al. [5] directly compared the effects of bone marrow-derived MSCs isolated from a 40-week fetus with the effects of bone marrow-derived MSCs isolated from an adult. The researchers found marked growth promotion from both cell types. However, the tumors injected with adult MSCs developed more rapidly (100% incidence of tumors by day 20) than did those injected with fetal MSCs (20%–80% tumor incidence by day 20), suggesting that the adult MSCs had greater tumor growth-promoting activity.

The effects of propagating cells ex vivo are also variable and may contribute to these discrepant results. Authors have reported growing MSC in high serum or growth factor supplemented media, which may alter the MSC phenotype. In addition, passage number and confluence may impact MSC function. Studies using endogenously labeled lineages of stromal cells will eliminate these concerns and will help clarify the effects of MSC or MSC-like cell in vivo. Finally, it is not known what proportion of “MSC” cell lines are contaminated with tumor cells, such as reported contamination with osteosarcoma and glioma cells used in the same laboratories [15]. Fingerprinting of all cell lines used should therefore be de rigueur and will help reducing confusion regarding biological properties of MSC.

In vivo tumor models vary widely and may also account for some of the differences in the findings of these studies. Tumor immunity, hypoxia, angiogenesis, and cytokine secretion are all highly variable in the models described in these studies. However, MCF-7 breast cells and B16 melanoma cells were reported by different investigators to be both promoted and suppressed by MSC delivery, suggesting that the tumor model is not the only factor accounting for the disparate findings.

The dose of MSCs delivered has been proposed to be a determinate of MSC effects on tumor growth. In general, studies that reported growth promotion tended to use a higher MSC to tumor cell ratio. However, low percentages of MSCs, such as the 10% used by Yu et al. [9], promoted subcutaneous growth in a lung and glioma cell line. These researchers [49] reported that coinjection of a higher percentage of MSCs further augmented tumor growth, suggesting that, in the right conditions, MSCs may impact tumor growth in a dose-dependent manner.

The timing at which MSCs are introduced into the tumor microenvironment may be an important consideration. All three of the studies that introduced MSCs into established tumors reported tumor growth inhibition [21, 20, 23]. Three other studies reporting growth inhibition involved modifications, such as implantation into a gelatin matrix or intravenous delivery of MSCs, which may have minimized the direct contact of MSCs during tumor initiation. By contrast, all of the studies reporting growth promotion mixed MSCs with tumor cells and coinjected the cells. The presence of MSCs during early tumor growth may facilitate processes, such as angiogenesis, which are required for tumor initiation. This hypothesis is consistent with the studies investigating MSC interactions with the vasculature. Coinjection of MSCs with pancreatic cancer cells resulted in increased vessel density, which required MSC-derived VEGF expression [47]. By contrast, MSCs induced endothelial cell apoptosis in established Matrigel capillaries, and delivery of MSCs into established melanoma xenografts abrogated tumor growth.

Finally, patient-to-patient variability in MSC isolates may also contribute to the conflicting data reported in the literature. It is possible that due to genetic, epigenetic, or environmental effects, MSCs may have different effects on the tumor microenvironment and could conceivably contribute to an individual's cancer risk. To address this, studies of MSCs from patients predisposed to cancer because of somatic mutations, such as BRCA, or environmental exposures, such as cigarette smoking, may be helpful to determine if patient-to-patient variability exists.

In summary, no simple paradigm can account for the conflicting findings in the studies of MSCs. Understanding the nuances of the interaction between MSCs and cancer cells is especially critical given the therapeutic potential of MSCs. The possibility of MSCs promoting tumor growth and metastasis raises concerns about the safety of their use as clinical tools. No evidence of tumor formation has been reported in over 1,000 patients treated with MSC for a variety of indications, so far. MSCs that have been engineered to express antitumor cytokines have potent antitumor effects, suggesting that perhaps putative tumor-promoting effects of MSCs can be overcome by manipulating cytokine expression. Additionally, MSCs could also be engineered to express a suicide gene so that MSCs delivered systemically would migrate into tumors, express an antitumor protein, and then be terminated with the delivery of an agent, such as gancyclovir, resulting in selective termination of the exogenously delivered MSCs. Furthermore, therapeutic strategies aimed at disrupting tumor-promoting effects of MSCs opens the door to a novel therapeutic strategy, but this would need to be properly timed to prevent disruption of a growth-suppressive MSC-tumor cell interaction. This review highlights the critical need for studies designed to explicitly test the hypothesis that tissue source, individual donor variability, timing of MSC injection, or expression of critical receptors such as TLR, determine the effect of MSC on tumor progression. Only systematic analysis of the role of these and other factors will resolve this important issue.
